# Supplementary material for: Fractional order SEIQRD epidemic model of Covid-19: A case study of Italy
Source: PLoS One. 2023 Mar 6;18(3):e0278880. doi: 10.1371/journal.pone.0278880 (PMC9987810; doi:10.1371/journal.pone.0278880)
Supplement: Attachment — Submitted filename: Reviewer Report [PONE-D-22-16861].pdf [file pone.0278880.s001.pdf]

Reviewer Report on:

Title: *Fractional order SEIQRD epidemic model of Covid-19: a case study of Italy*

Journal: *PLOS ONE*

Manuscript ID: *PONE-D-22-16861*

Authors: *Mahata et al.*

Overall Comments:

The authors have examined the fractional order SEIQRD compartmental model in a fractional order framework to account for the uncertainty caused by the lack of information regarding the Coronavirus (COVID-19). In addition, the fractional-order Taylor's approach is utilized to approximate the solution to the proposed model. The model's validity is demonstrated by comparing real-world data with simulation outcomes. Furthermore, the system's analysis and numerical findings show that regular usage of face masks can help to reduce the COVID-19 epidemic.

However, it needs some revisions from the point of authors and readers to improve the quality of the paper. After these **MAJOR REVISIONS**, I suggest that this paper can be accepted to publish in " ***PLOS ONE***".

My comments are as follows:

1. Ensure the end of each line of the equations has a punctuation, either a comma or a full stop if it is the end of the equation - these are missing in several of the equations, see for example:

$$^{C}D_t^{\phi}(u(t)) = \frac{1}{\Gamma(n-\phi)}\int_0^t \frac{1}{(t-z)^{\phi+1-n}}\frac{d^n}{dz^n}u(z)dz$$

where $C^n\,[0,\infty]$ is a n tines continuously differentiable function and the Gamma function is defined by $\Gamma(\ \ )$such that $n-1<\phi<n$".

Lemma 2 " Let us consider the fractional order system as

$$^{C}D_t^{\phi}(Y(t)) = \Psi(Y), Y_{t_0} = \left(y_{t_0}{}^1, y_{t_0}{}^2, \dots, y_{t_0}{}^n\right), y_{t_0}{}^j, j = 1,2,\dots,n$$

with $0<\phi<1, Y(t) = (y^1(t), y^2(t), \dots, y^n(t))$ and $\Psi(Y) : [t_0,\infty] \to \mathbb{R}^{n\times n}$. For calculate the equilibrium points, we have $\Psi(\mathrm{Y}) = 0$. These equilibrium points are locally asymptotically stable

2. The Introduction should make a compelling case for why the study is useful along with a clear statement of its novelty or originality by providing relevant information and providing answers to basic questions such as:

   - What is already known in the open literature?
   - What needs to be done, why and how?

3. Clear statements of the novelty of the work should also appear briefly in the Abstract and Conclusions sections.

4. How did they decide the Lyapunov function in the global stability analysis?

5. How did they decide determining the initial conditions?
6. What about the parameter values in the system. Where they have been taken them from?
7. Whay did they ignore the fractional order in the system? Is it dimentionally correct now? Discuss the system by comparing the following literature.

$$
\begin{aligned}
{}^{C}D_t^{\phi}\, S(t) &= a - b(1-\alpha\beta)S(t)I(t) - c\, S(t),\\
{}^{C}D_t^{\phi}\, E(t) &= b(1-\alpha\beta)S(t)I(t) - (e + c)\, E(t),\\
{}^{C}D_t^{\phi}\, I(t) &= e\, E(t) - (f + g + h + c)\, I(t),\\
{}^{C}D_t^{\phi}\, Q(t) &= f\, I(t) - (k + l + c)\, Q(t),\\
{}^{C}D_t^{\phi}\, R(t) &= g\, I(t) + k\, Q(t) - c\, R(t),\\
{}^{C}D_t^{\phi}\, D(t) &= h\, I(t) + l\, Q(t).
\end{aligned}
\qquad (3.3)
$$

- Fractional order modelling of omicron SARS-CoV-2 variant containing heart attack effect using real data from the United Kingdom. Chaos, Solitons & Fractals, 111954, (2022).
- Investigation of interactions between COVID-19 and diabetes with hereditary traits using real data: A case study in Turkey. Computers in biology and medicine, 105044, (2021).

8. What is the main motivation in using the Caputo operator? What is the main advantages of it on other existing operators with or without singular kernels?
9. The authors are requested to add more details regarding their original contributions in this manuscript.
10. Some figures should be redrawn clearly.
11. Authors should improve the introduction by including the recent development within the frame of COVID-19 pandemic and its variants with the help of recently published papers. I recommend the papers:
    - Modeling and analysis of COVID-19 epidemics with treatment in fractional derivatives using real data from Pakistan. The European Physical Journal Plus, 135(10), 1-42, (2020).
    - Stability analysis of an incommensurate fractional-order SIR model. Mathematical Modelling and Numerical Simulation with Applications, 1(1), 44-55, (2021).
    - A new mathematical modeling of the COVID-19 pandemic including the vaccination campaign. Open Journal of Modelling and Simulation, 9(3), 299-321, (2021).
    - Vaccination effect conjoint to fraction of avoided contacts for a Sars-Cov-2 mathematical model. Mathematical Modelling and Numerical Simulation with Applications, 1(2), 56-66, (2021).
    - Extinction and stationary distribution of a stochastic COVID-19 epidemic model with time-delay. Computers in Biology and Medicine, 105115, 141, (2022).
    - Dynamics of cholera disease by using two recent fractional numerical methods. Mathematical Modelling and Numerical Simulation with Applications, 1(2), 102-111, (2021).
    - Chaos of calcium diffusion in Parkinson's infectious disease model and treatment mechanism via Hilfer fractional derivative. Mathematical Modelling and Numerical Simulation with Applications, 1(2), 84-94, (2021).
    - Fractional-order mathematical modelling of cancer cells-cancer stem cells-immune system interaction with chemotherapy. Mathematical Modelling and Numerical Simulation with Applications, 1(2), 67-83, (2021).

12. What is the novelty of your work? There are some similar papers which investigated in this area, although there are some minor differences in the structure and applications to the COVID-19 models. Please state it clearly.

13. Have you employed any specific assumptions in your COVID-19 model? Please explain briefly.

14. More biological interpretations should be given. Please provide corresponding explanations of the figures in terms of their biological meanings and the pointing out the novelty of the paper. How do figures support your scheme? It will be more helpful to readers to have some discussions about insight of the main results and outcomes of the figures.

Briefly, I recommend publishing after doing above **MAJOR REVISIONS**. So, I want to read speedily the revised version of paper before publishing if it is possible for you.

With many thanks and best regards…
